# Alpha Adrenergic Induction of Transport of Lysosomal Enzyme across the Blood-Brain Barrier

**DOI:** 10.1371/journal.pone.0142347

**Published:** 2015-11-06

**Authors:** Akihiko Urayama, Shinya Dohgu, Sandra M. Robinson, William S. Sly, Jeffery H. Grubb, William A Banks

**Affiliations:** 1 Department of Neurology, University of Texas Medical School at Houston, Houston, TX, United States of America; 2 Department of Pharmaceutical Care and Health Sciences, Faculty of Pharmaceutical Sciences, Fukuoka, Japan; 3 Division of Geriatric Medicine, Department of Internal Medicine, Saint Louis University, St. Louis, MO, United States of America; 4 Edward A. Doisy Department of Biochemistry and Molecular Biology, Saint Louis University School of Medicine, St. Louis, MO, United States of America; 5 Geriatric Research, Education, and Clinical Center, Veterans Affairs Puget Sound Health Care System, Seattle, WA, United States of America; 6 Division of Gerontology and Geriatric Medicine, Department of Medicine, University of Washington School of Medicine, Seattle, WA, United States of America; Hungarian Academy of Sciences, HUNGARY

## Abstract

The impermeability of the adult blood-brain barrier (BBB) to lysosomal enzymes impedes the ability to treat the central nervous system manifestations of lysosomal storage diseases.

Here, we found that simultaneous stimulation of the alpha1 and alpha2 adrenoreceptor restores in adult mice the high rate of transport for the lysosomal enzyme P-GUS that is seen in neonates but lost with development. Beta adrenergics, other monoamines, and acetylcholine did not restore this transport. A high dose (500 microg/mouse) of clonidine, a strong alpha2 and weak alpha1 agonist, was able to act as monotherapy in the stimulation of P-GUS transport. Neither use of alpha1 plus alpha2 agonists nor the high dose clonidine disrupted the BBB to albumin. *In situ* brain perfusion and immunohistochemistry studies indicated that adrengerics act on transporters already at the luminal surface of brain endothelial cells. These results show that adrenergic stimulation, including monotherapy with clonidine, could be key for CNS enzyme replacement therapy.

## Introduction

Lysosomal storage diseases (LSDs) are autosomal resessive disorders characterized by inherited deficiency in lysosomal metabolic activity. The lack of various acid hydrolases constitutes more than 50 different diseases, each disease having a specific deficiency of a lysosomal enzyme. Enzyme replacement therapy (ERT) has been very effective in treating several LSDs, including mucopolysaccharidoses [1, 2]. While ERT by the intravenous route effectively ameliorates abnormal storage in peripheral organs, correcting central nervous system (CNS) storage has been challenging due to the blood-brain barrier (BBB) hampering the entry of lysosomal enzymes from the blood to brain. For this reason, several approaches are being developed to improve enzyme delivery to the brain, including antibody-directed delivery, improved pharmacokinetics, intrathecal delivery, and targeting of brain endothelial cell transporters [3–5].

Brain microvessel endothelial cells composing the BBB retain several non-specific or specific mechanisms for transcellular transport of macromolecules, including the receptor-mediated, adsorptive-mediated, fluid-phase micropinocytosis, and macropinocytosis [6]. Earlier studies showed that circulating macromolecules appeared in micropinocytic vesicles within the endothelial cells, that micropinocytic vesicles fused with lysosomes, and that macromolecules not resistant to lysosomal degradation were not found beyond the vascular endothelial cell linings [7, 8]. These observations suggested that lysosomes were involved in transcellular transport of lysosomal enzymes in brain endothelial cells, just as they are involved in lysosomal enzyme intracellular trafficking in other types of endothelial cells [9]. Another mechanism for macromolecule transport across the BBB is that of macropinocytosis; the existence of this pathway is lately suggested in human brain microvessel endothelial cells [10, 11].

In our prior studies, we found that the cation-independent mannose 6-phosphate (M6P) receptor participates in brain uptake of systemically circulating lysosomal enzymes across the neonatal BBB [12, 13]. Developmental down-regulation of this receptor-mediated uptake mechanism resulted in failure of brain delivery of lysosomal enzyme across the adult BBB. We postulated that the inability of this transport mechanism in the adult BBB is from the loss of cell surface M6P receptor, while the receptor remains in the intracellular pool. Eventually, we found that M6P receptor-mediated transcytosis of lysosomal enzymes across the BBB was restored by epinephrine in adult mice [14], suggesting that the adrenergic effects of epinephrine modify the transcytotic activity mediated through the M6P receptor which participates in the cellular trafficking of lysosomal enzymes.

The regulatory mechanisms involved in the re-induction of the M6P receptor transport of lysosomal enzymes across the BBB by epinephrine remain to be elucidated. Currently, there is no direct evidence that epinephrine modulates the activity of the M6P receptor itself. Brain microvessel endothelial cells express both α- and β-adrenoceptors [15]. Both adrenoceptors, including receptor subtypes of each, can initiate the internalization of receptors, inducing their redistribution from the cell surface to cytoplasmic vesicles [16–18]. The biological role of receptor internalization may have a variety of spatio-temporal effects, possibly including effects on the redistribution of endosomal M6P receptors. The present study addresses the regulatory mechanisms by which the adrenergic system modulates the transport of P-GUS across the adult BBB by employing series of receptor agonists and antagonists in vivo.

## Materials and Methods

### Production of PGUS

PGUS was produced in overexpressing, cation-independent M6P receptor-deficient mouse L cells as described previously [4]. The enzyme was purified from conditioned media by anti-human GUS mAb affinity column chromatography. PGUS was eluted with 3.5 M MgCl_2_, then desalted over Bio Gel P6 sizing resin (Bio-Rad, Hercules, CA). The concentration of PGUS was adjusted to 2.5 × 10^5^ units per ml (1 unit = 1 nmol of substrate cleaved per h) and the purified enzyme was stored at -70°C. M6P-specific uptake of the PGUS by human fibroblasts was 185 units per mg/h (data not shown).

### Radioactive labeling

PGUS was radioactively labeled using the iodobead method (Pierce, Rockford, IL) with [^131^I]Na (Perkin Elmer, Waltham, MA). The use of a single iodobead allows controlled iodination, so that both enzymatic activity and susceptibility to endocytosis by the M6P receptor are preserved [12–14]. Labeled, active enzyme (I-PGUS) was separated from free iodine on a Sephadex G-10 column. Albumin was labeled with [^125^I]Na (Perkin Elmer) using the chloramine-T method, and purified (I-Alb) on a column of Sephadex G-10. Each reagent was freshly prepared on the day of the experiment.

### Animals

Adult male CD-1 mice from our in-house colony were studied at 7–8 weeks of age. The mice had free access to food and water and were maintained on a 12-hour dark/light cycle in a room with controlled temperature. All the animal studies were approved in advance by the St. Louis VA Institutional Animal Care and Use Committee, conducted according to national and international guidelines, and carried out at the VA which is a facility that is approved by the Association for Assessment and Accreditation of Laboratory Animal Care.

### Epinephrine Effects on I-PGUS influx and BBB disruption

Multiple-time regression analysis was used to measure blood-to-brain uptake of I-PGUS and I-Alb [19, 20]. Disruption of the BBB was measured as an increase in the brain/serum ratio for I-Alb and the unidirectional influx rate of I-PGUS was measured after correction for the I-Alb space. Adult male CD-1 mice were anesthetized with an I.P. injection of urethane (40%) and a 200 μl injection of lactated Ringers solution containing1% BSA and 5.5x10^5^ cpm each of I-PGUS or I-Alb was injected into the jugular vein with or without 40 or 120 nmol/injection of epinephrine. Brain and carotid artery blood samples were obtained 1, 2, 3, 4, 5, 6, 7.5, and 10 min after the iv injection (n = 3-4/time point), arterial blood was collected from the carotid artery and the brain was removed and weighed. Levels of radioactivity in the brain and 50 μl of serum were counted in a gamma counter for 3 minutes. Brain/serum ratios were calculated to yield units of μl/g and plotted against exposure time. The linear portion of the slope of the resulting correlation measures the unidirectional influx rate in units of μl/g-min and the Y-intercept measures the distribution space in brain at t = 0 in units of μl/g. The effect of epinephrine on blood clearance and volume of distribution were also measured by plotting the percent of the injected dose found per ml of serum (%Injected dose/ml) against time.

### Monoamines, Acetylcholine, and Adrenergic Agents

All reagents were purchased from Sigma-Aldrich Chemical Co (St Louis MO) unless otherwise noted. Reagents were dissolved in lactated Ringers solution with 1% by weight of bovine serum albumin unless otherwise noted. Concentrations are given in moles/mouse except when the literature has primarily given doses in g/mouse, in which case moles are given in parentheses. Adult male mice anesthetized with urethane were given an injection into the jugular vein of lactated Ringer’s solution containing 1% BSA and 4x10^5^ cpm of both I-PGUS and I-Alb with or without an agent. For antagonists, 40 nmol/mouse of epinephrine was included in the iv injection. After 10 min, blood was obtained from the carotid artery and the brain removed. Serum derived from the arterial blood and the whole brain were counted in a gamma counter for 3 min and the results expressed as brain/serum ratios in units of μl/g. Acetylcholine and the monoamines dopamine, histamine, and serotonin were tested at the dose of 200 nmol/mouse. General adrenergic receptor antagonists tested were propranolol (general beta) 200 nmol/mouse, yohimbine (alpha2) 100 nmol/mouse, phentolamine (general alpha) 200 nmol/mouse, and prazocin (alpha1) 100 nmol/mouse. Antagonists that were more selective for their sites were benoxathian (specific alpha1) 250 μg/mouse (628 nmol/mouse) and CGP20712A (specific beta) 250 μg/mouse (420 nmol/mouse). General agonists tested were isoproterenol (general beta agonist) 10 nmol/mouse, L-phenylephrine (alpha1) 10 nmol/mouse, and clonidine (alpha2) 10 nmol/mouse. Agonists that were more selective for their sites were UK 14,304 with it and its control tested in 14.9% DMSO (alpha2) 200 μg/mouse (682 nmol/mouse), albuterol (selective beta) 200 μg/mouse (694 nmol/mouse), and cirazoline (alpha1) 100 μg/mouse (463 nmol/mouse) In follow-up studies, cirazoline 100 μg/mouse plus clonidine 200 μg/mouse were also tested and clonidine alone was tested at 50, 200, and 500 μg/mouse.

### Immunohistochemistry in brain endothelial cell monolayers

Primary culture of mouse brain endothelial cells were isolated from adult male CD-1 mice (8 weeks old) as previously described [21]. Brain microvessel endothelial cells were treated with or without epinephrine (10 μM) in serum-free DMEM/F-12 (Sigma) supplemented with 100 units/mL penicillin, 100 μg/mL streptomycin, 50 μg/mL gentamicin, 2 mM GlutaMAX^™^-I and 1 ng/mL basic fibroblast growth factor (bFGF; Sigma) for 30 min. After fixation with 3.7% formaldehyde (Sigma) for 10 min at room temperature, they were incubated with anti-M6P receptor antibody (Abcam) in 1% BSA/PBS for overnight at 4°C. Then, they were washed once with PBS, three times with balanced salt solution (130 mM NaCl, 5.4 mM KCl, 1.8 mM CaCl_2_, 4 mM MgCl_2_, 20 mM HEPES, 5.5 mM glucose, pH 7.4) and once with PBS, and incubated with 20 μg/mL Alexa Fluor 488-conjugated anti-mouse IgG (Invitrogen) in 1% BSA/PBS for 1 hr at room temperature. After washing, cells were covered with Vectashield Hard Set mounting medium (Vector Laboratories, Burlingame, CA) and coverslips were applied. Fluorescence was detected with Zeiss Axiovert 40 CFL fluorescent microscope.

### *In situ* transcardiac brain perfusion

Adult male CD-1 mice were anesthetized with 40% urethane, the heart exposed, both jugulars severed, and the descending thoracic aorta ligated. A 26-gauge butterfly needle was inserted into the left ventricle of the heart, and the perfusion fluid (7.19 g/liter NaCl, 0.3 g/liter KCl, 0.28 g/liter CaCl_2_, 2.1 g/liter NaHCO_3_, 0.16 g/liter KH_2_PO_4_, 0.17 g/liter anhydrous MgCl_2_, 0.99 g/liter d-glucose, and 1% wt/vol BSA), containing 10^5^ cpm each of I-PGUS and I-Alb with or without 1 nmol/ml or 10 nmol/ml of epinephrine was infused at a rate of 2 ml/min for 5 min. The perfusion fluid was freshly prepared each day. After perfusion, the whole brain was removed and weighed. The level of radioactivity was determined in a γ-counter.

### Statistical Analysis

Means are reported with their standard error terms. Statistical comparisons were made between two groups using Student’s t-test. More than two groups were compared by analysis of variance (ANOVA) with Newman-Keuls used as the post-test. Regression lines were computed by the least squares method and the slopes and intercepts compared using the statistical package in Prism 5.0 (GraphPad, Inc San Diego, CA).

## Results

### Epinephrine on I-PGUS influx and BBB disruption

**Fig 1:**
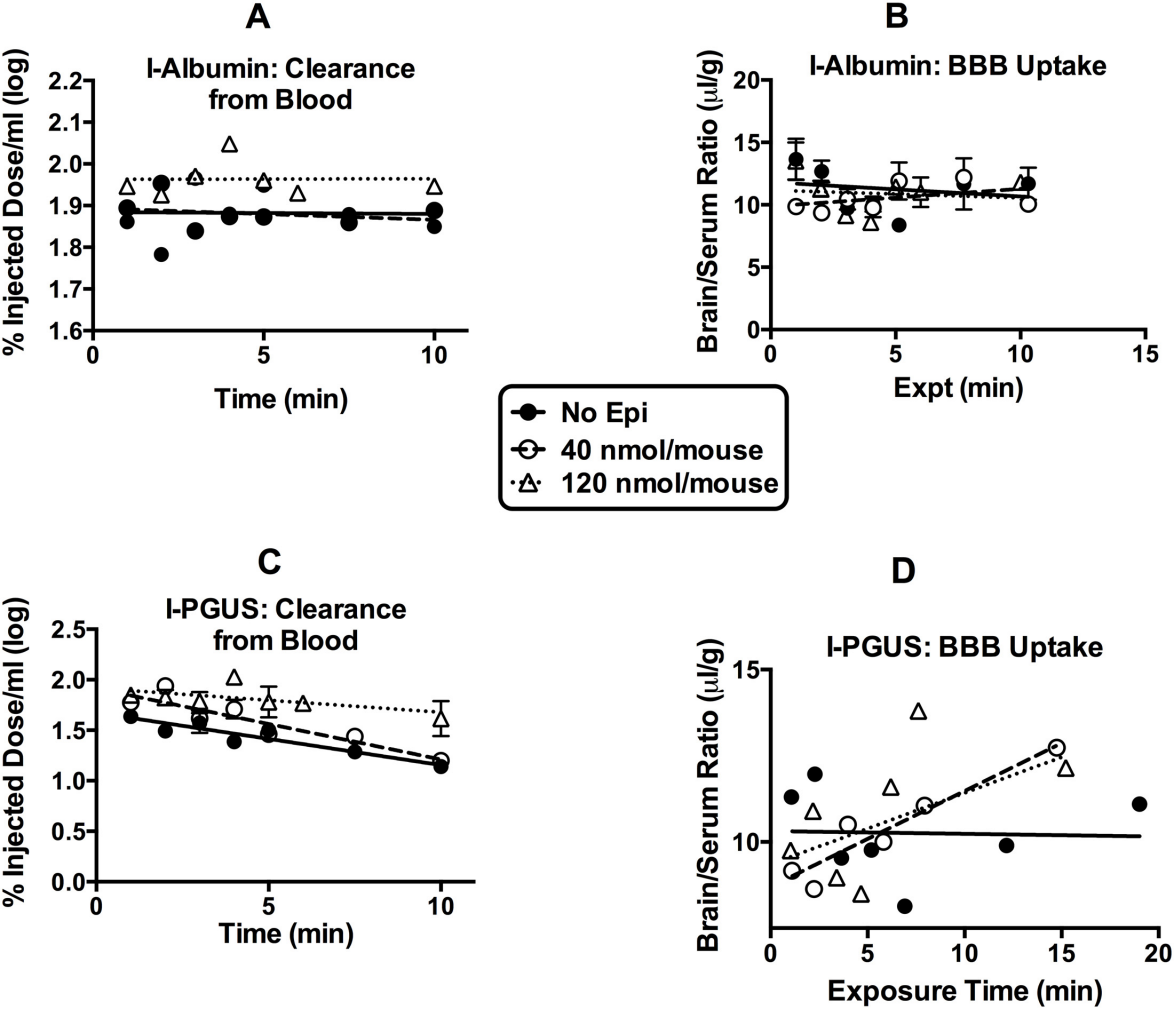
Blood-brain barrier disruption and brain pharmacokinetics of PGUS at two doses of epinephrine. (A) shows that the 120 nmol/mouse dose of epinephrine decreased the vascular space of I-Alb. (B) shows that neither dose disrupted the BBB. (C) shows that both doses reduced vascular space of I-PGUS and that the 120 nmol/mouse dose also slowed clearance from blood. (D) shows that I-PGUS did not cross the BBB, but that both doses of epinephrine induced transport of I-PGUS across the BBB. n = 7/group.

Fig 1 shows the serum level-time profiles (panels A and C) and the brain/serum ratios (panels B and D) of I-albumin and I-PGUS after i.v. injection with epinephrine. Epinephrine at the 120 nmol, but not the 40 nmol/mouse dose, slightly decreased the distribution space for I-Albumin (F(2,61) = 6.3, p<0.01) in the systemic circulation. Systemic volumes of distribution decreased by about 16% from 1.3 ml to 1.1 ml, suggesting leakiness of the peripheral blood vessels. Both doses of epinephrine decreased the systemic volume of distribution (Panel C) for I-PGUS and the 120 nmol/mouse dose also decreased clearance from blood. The brain/serum ratios of I-albumin (Panel B) shows that, in this experiment, neither dose of epinephrine disrupted the BBB. However, the ratios of I-PGUS (Panel D) shows that both doses were equally effective in increasing the uptake by brain of I-PGUS (F(2,52) = 4.65, p<0.05) from cerebral circulation.

### Monoamines, Acetylcholine, and Adrenergic Agents

**Fig 2:**
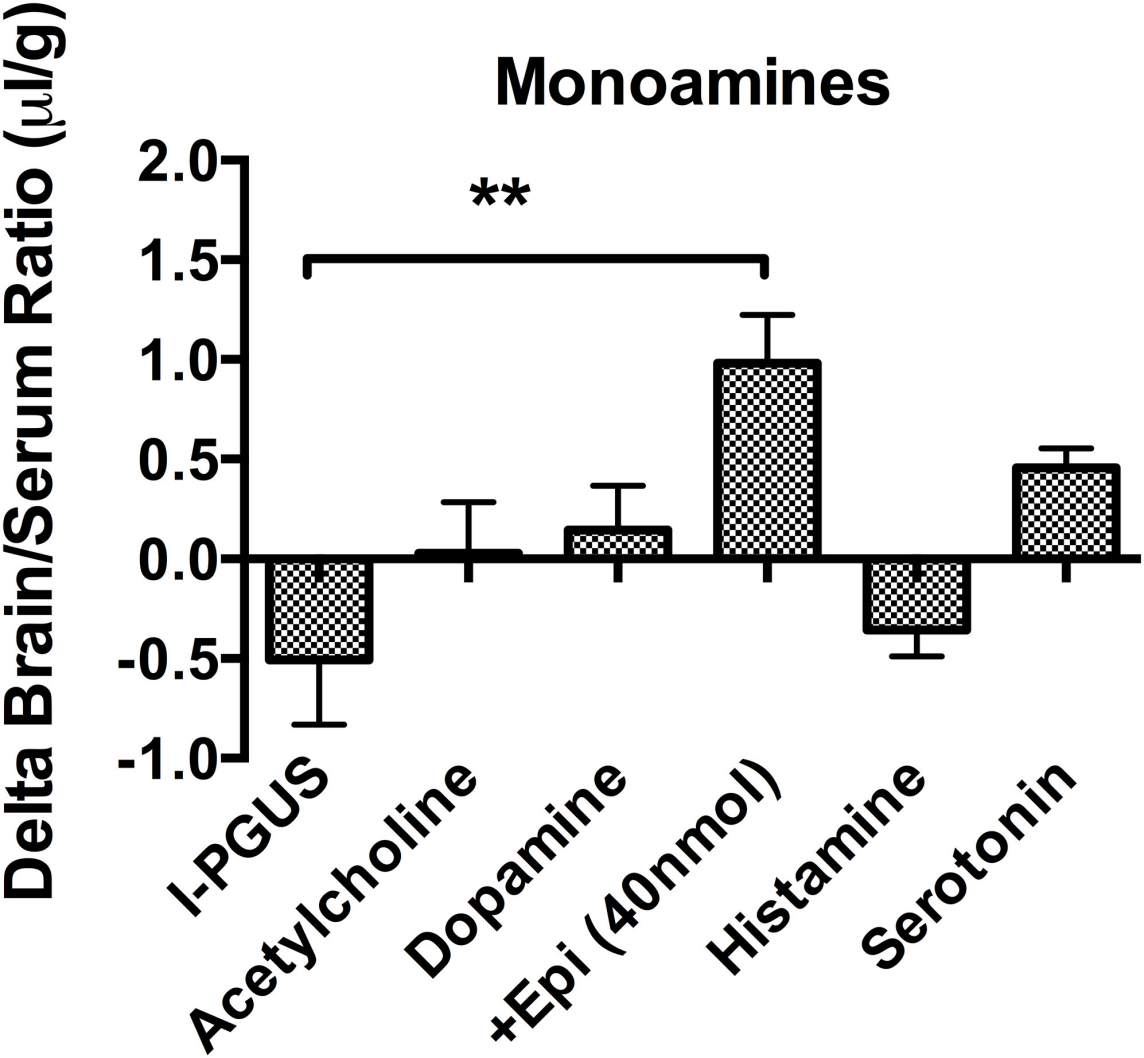
Effect of monoamines and acetylcholine on I-PGUS uptake. **Only epinephrine enhanced BBB penetration.** **P<0.01; n = 7-10/group.

**Fig 3:**
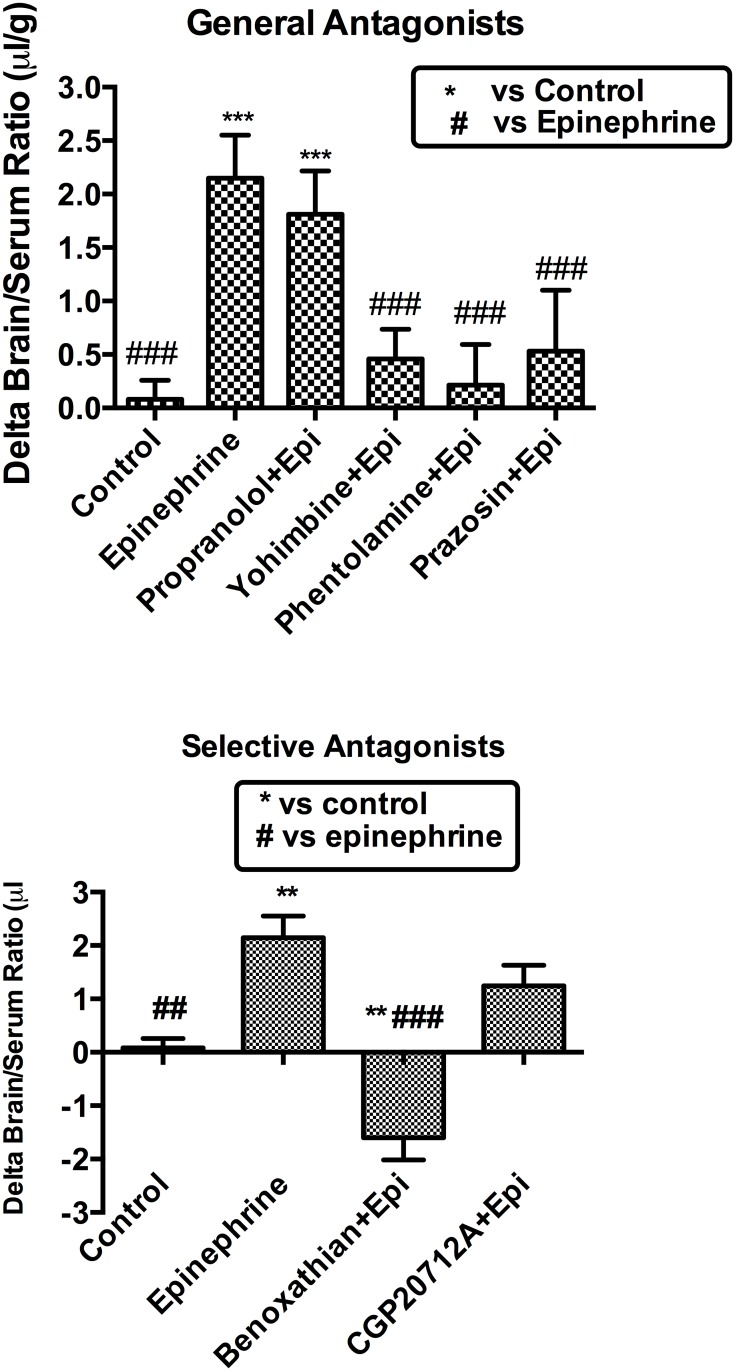
Effect of adrenergic antagonists on epinephrine-induced uptake of I-PGUS across the BBB. Upper panel shows that more general alpha, alpha1, and alpha2 antagonists but not beta antagonists blocked epinephrine’s effect. Lower panel shows results that the highly selective alpha1 adrenergic receptor antagonist benoxathian but not the highly selective beta adrenergic receptor antagonist CPG20712A blocked the effect of epinephrine. **p<0.01 and ***p<0.001 inc comparison to control; ##p<0.01 and ###p<0.001 in comparison to epinephrine.

**Fig 4:**
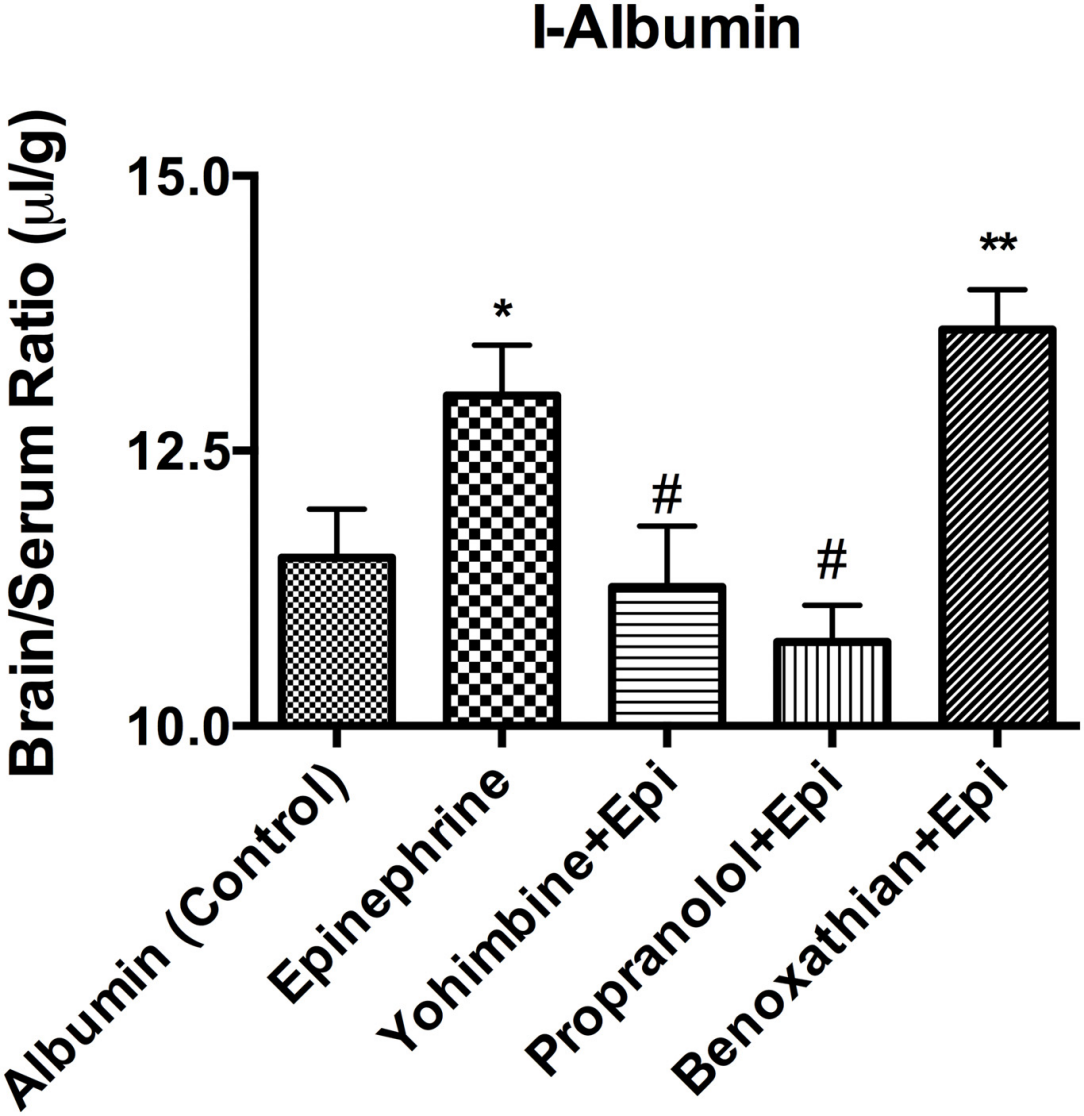
Effects of adrenergic antagonists on epinephrine-induced BBB disruption. Blockade of alpha2 (yohimbine) and beta (propranolol) but not of alpha1 (benoxathian) receptors prevented epinephrine-induced BBB disruption. *p<0.05 in comparison to Control; **p<0.01 in comparison to control; #p<0.05 in comparison to epinepthrine; n = 8/group.

We investigated the regulatory mechanism of neurotransmitters on I-PGUS transport across the BBB by employing various combinations of agonists and antagonists for adrenergic, serotonergic, muscarinic, and histaminergic receptors. Fig 2 shows that effects of monoamines on brain uptake of I-PGUS 10 min after i.v. injection. The uptake was evaluated by the brain/serum ratio with the contribution of the vascular space as measured with I-albumin subtracted. We found that simultaneously administered acetylcholine, dopamine, histamine, and serotonin were without effect on the BBB penetration of I-PGUS, suggesting the increased brain uptake of I-PGUS was epinephrine specific. We then tested adrenoceptor antagonists to determine their abilities to block the effect of epinephrine (Fig 3). We found that the epinephrine-induced increase in I-PGUS uptake by brain was inhibited by the non-specific alpha antagonist phentolamine (200 nmol), the alpha1 selective antagonist prazosine, and the alpha2 selective antagonist yohimbine (100 nmol), but not by the beta antagonist propranolol (200 nmol). Benoxanthian, a selective alpha1 antagonist, blocked the epinephrine effect, whereas CGP20712A, selective beta1 antagonist, did not. These pharmacologic inhibitions suggest that epinephrine induced brain uptake of P-GUS was mediated through alpha receptors, but not by beta receptors. In this set of experiments, epinephrine did disrupt the BBB to I-Albumin (Fig 4). This epinephrine-induced BBB disruption was significantly blocked by the alpha2 antagonist yohimbine and by the beta antagonist propranolol, but not by the alpha1 antagonist benoxathian.

**Fig 5:**
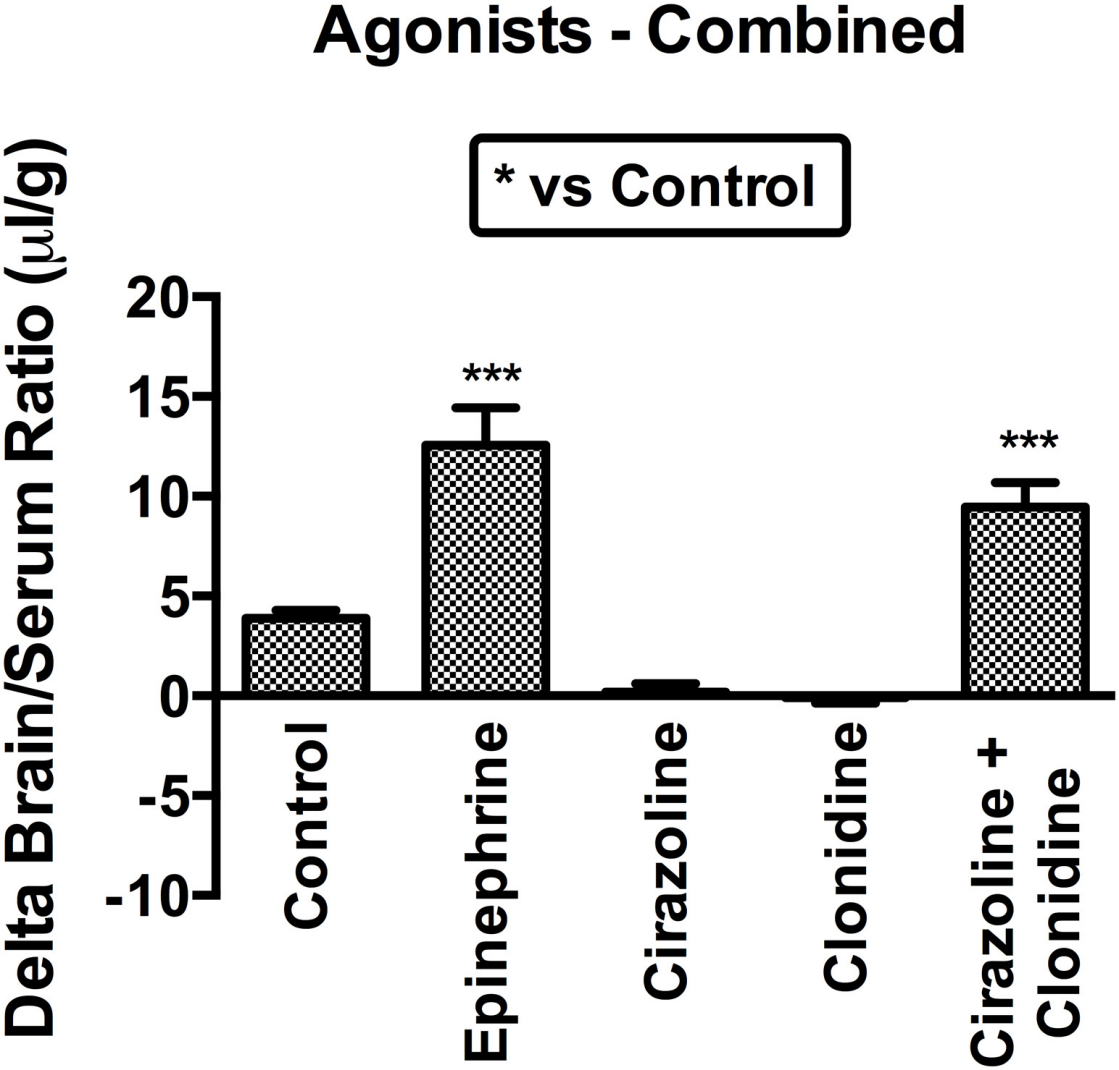
Combined effect of alpha1 (cirazoline) and alpha2 (clonidine) adrenergic receptor agonists on I-PGUS transport across the BBB. Neither drug alone affected I-PGUS uptake, but combined they effectively increased transport across the BBB. ***p<0.001 in comparison to control; n = 12/group.

**Fig 6:**
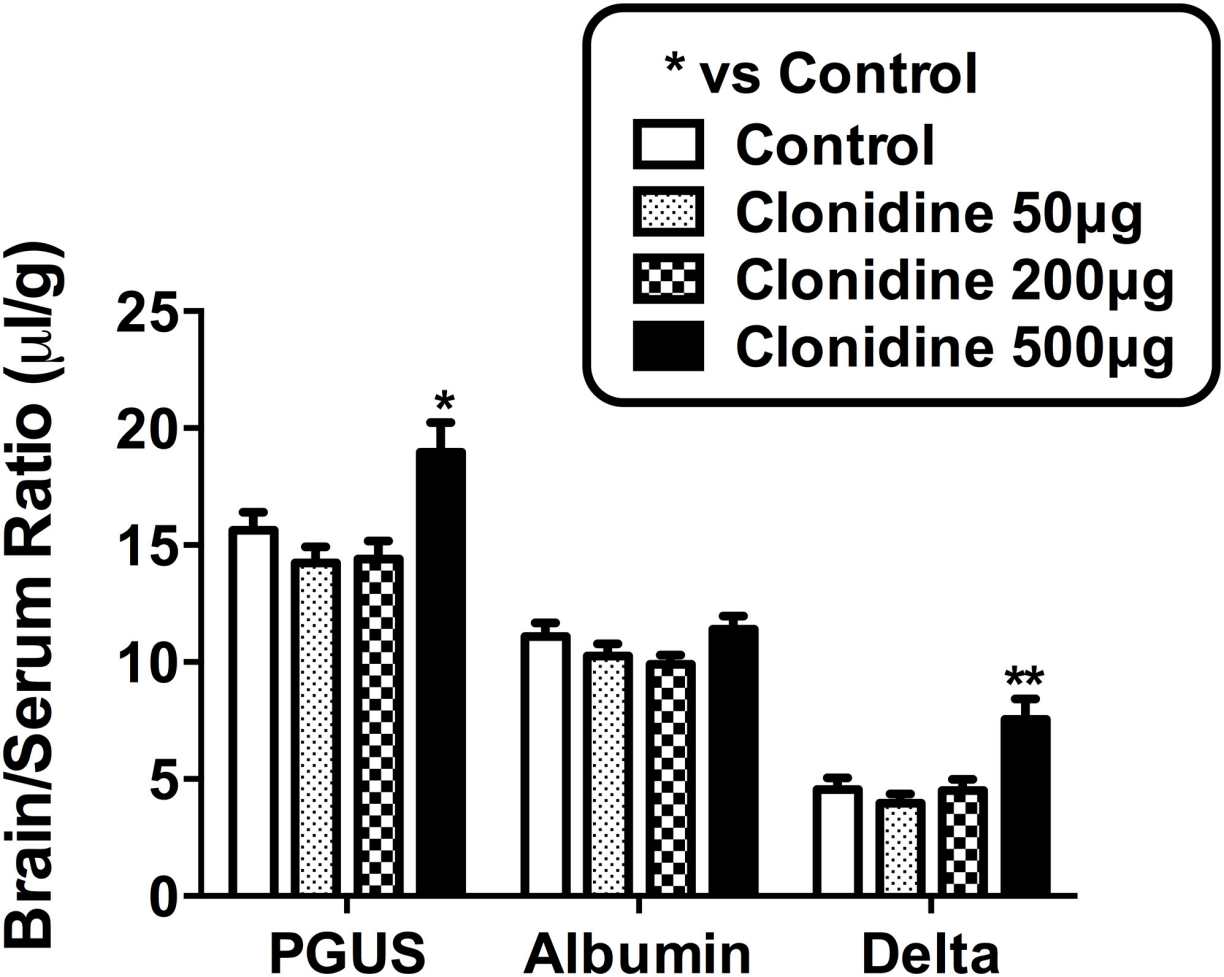
Dose response curve for clonidine: Effect on I-PGUS BBB transport. A high dose of clonidine enhanced I-PGUS transport without disrupting the BBB. *p<0.05 in comparison to control; n = 10/group.

Next, we examined general agonists for adrenergic receptors (Fig 5) and found that none of the alpha adrenergics agonists replicated the effects of epinephrine on I-PGUS BBB transport, including cirazoline and clonidine. However, combining the alpha1 agonist cirazoline (100 μg) and the alpha2 agonist clonidine (200 μg) increased I-PGUS to a level similar to that seen with epinephrine (Fig 5) without disruption of the BBB (data not shown). A high dose of clonidine (500 μg/mouse) was also effective in increasing I-PGUS BBB transport without disrupting the BBB (Fig 6). These results suggest that both apha1 and alpha2 receptor subtype activation is needed for the reinduction of transport for I-PGUS at the BBB.

### Immunohistochemistry in brain endothelial cell monolayers

**Fig 7:**
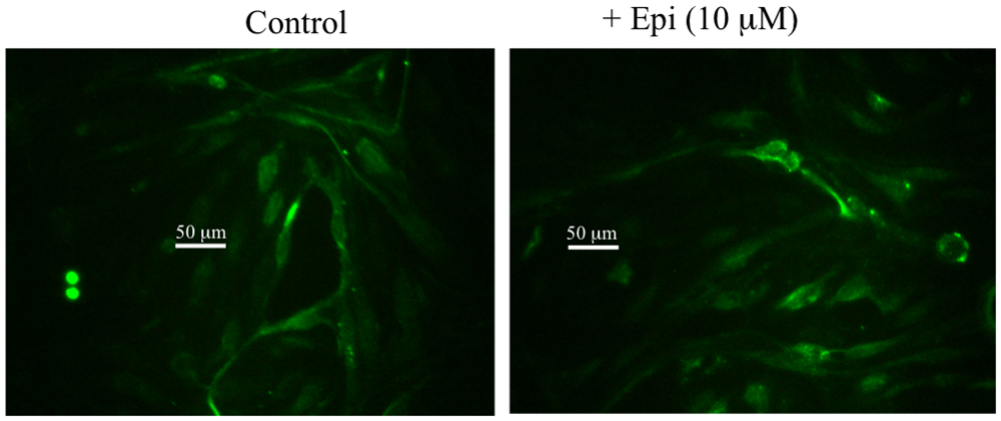
Expression of M6P receptor on nonpermeablized primary monolayer cultures of mouse brain endothelial cells. Receptor was detected on cells not treated with epinephrine and treatment did not enhance intensity of staining.

Fig 7 shows that non-permeablized monolayers of primary brain endothelial cells show the presence of M6P receptor on their cell surface. Addition of 10 μM of epinephrine did not change the intensity of staining, suggesting epinephrine-induced transport of I-PGUS across the BBB was mediated through existing M6P receptors, not by new expression of the receptor.

### *In situ* transcardiac brain perfusion

**Fig 8:**
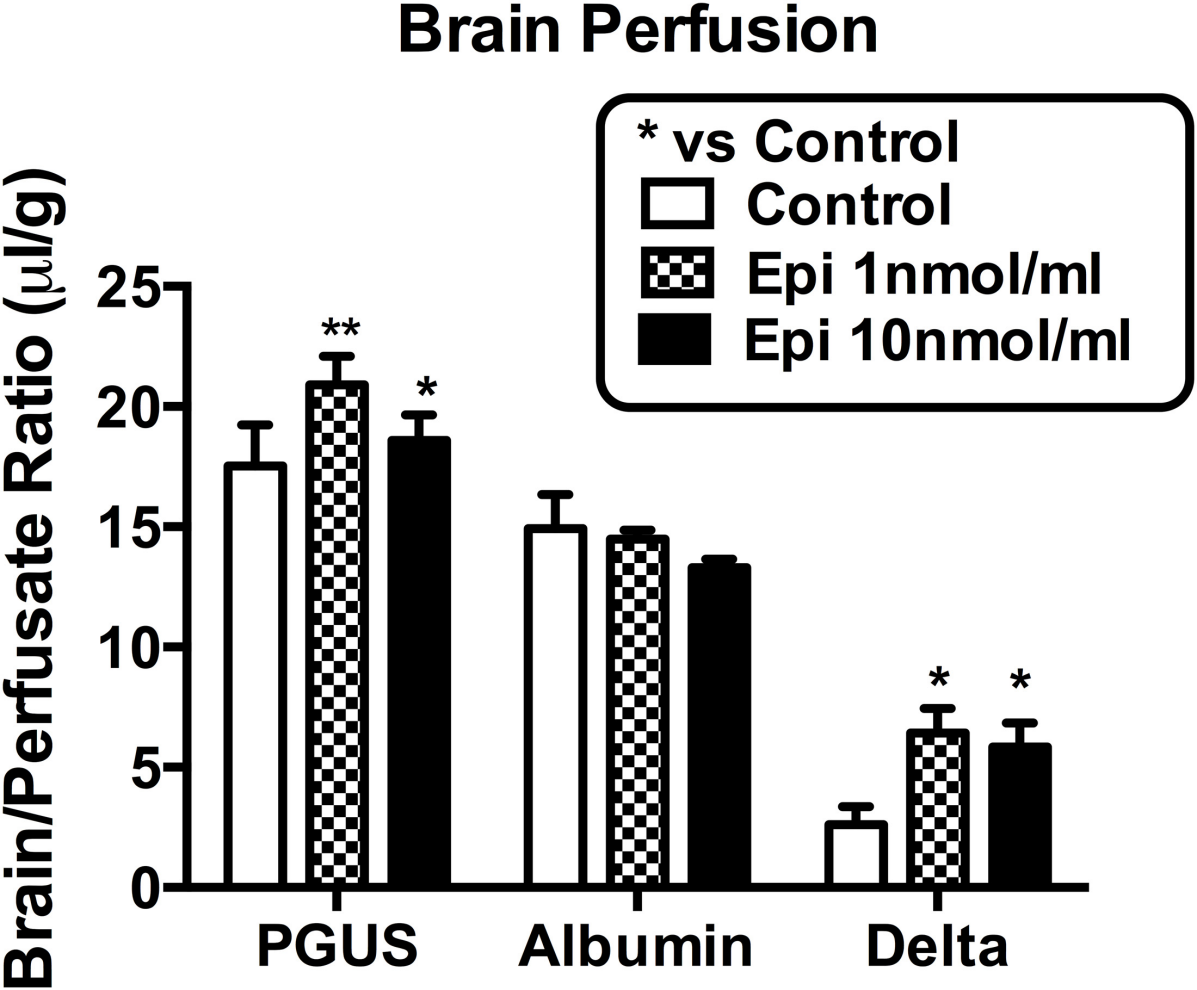
Brain perfusion of I-PGUS with or without epinephrine. Both doses of epinephrine enhanced BBB transport without disrupting the BBB. *p<0.05 and **p<0.01 in comparison to control; n = 8/group.

Epinephrine at both the 1 nmol/ml and the 10 nmol/ml doses were equally effective at increasing I-PGUS penetration (Fig 8). Neither dose disrupted the BBB to I-Alb. These data suggest that epinephrine acts directly at the BBB and not through peripheral factors.

## Discussion

Previous work has shown that many of the lysosomal enzymes given by peripheral administration, including PGUS, are taken up by the neonatal, but not the adult, brain [12, 13]. The BBB transporter responsible for the ability of PGUS to cross the neonatal BBB is the M6P receptor [12]. Previous work has also shown that epinephrine is able to restore M6P receptor activity at the BBB and the transport of lysosomal enzymes from blood to brain [14]. Fig 2 shows that this effect is selective to epinephrine as it is not shared with other monoamines or acetylcholine. Here, we further elucidate the mechanisms by which epinephrine facilitates PGUS transport across the BBB.

One mechanism by which epinephrine could facilitate blood-to-brain entry is by disrupting the BBB [22]. However, evidence here shows that this is not the case. First, the epinephrine-induced increase in I-PGUS uptake is consistent, whereas the epinephrine-induced disruption of the BBB as measured by I-Alb is sporadic. As such and as illustrated in Figs 1 and 6, I-PGUS is often seen to cross a BBB that is intact to I-Alb. As albumin is about 5 times smaller than PGUS, it is not conceivable that the BBB would be disrupted to PGUS and not also to albumin. Additionally as shown in Fig 4, epinephrine-induced disruption of the BBB is dependent on beta adrenergic stimulation, whereas epinephrine-induced stimulation of I-PGUS transport is dependent on alpha adrenergic stimulation.

The results with alpha adrenergic antagonists show that either an alpha1 or alpha2 antagonist can block epinephrine’s effect on I-PGUS transport, but neither an alpha1 nor an alpha2 agonist can replicate that effect. That led us to postulate that it might require simultaneous stimulation with both an alpha1 and alpha2 agonist to induce I-PGUS transport. We found that this was indeed the case in that whereas neither the alpha1 agonist cirazoline nor the alpha2 agonist clonidine was effective alone, in combination they replicated the effect of epinephrine (Fig 5). However, cirazoline is not an appropriate agent for clinical use because of toxicity. Clonidine is used clinically as an antihypertensive and, as it is a weak alpha1 agonist, we postulated that high doses of clonidine might stimulate both alpha1 and alpha2 receptors and so increase I-PGUS transport across the BBB. As shown in Fig 6, high doses of clonidine did indeed induce this effect. This underlines the possibility that adrenergics capable of stimulating both alpha1 and alpha2 receptors but not beta receptors could be administered with lysosomal enzymes to treat lysosomal storage diseases. Whether the dose of clonidine needed to do this in humans would also induce hypotension is not clear.

The rapid onset of the effect of epinephrine on I-PGUS transport across the BBB strongly suggests that epinephrine is not stimulating expression of the M6P receptor but rather activating receptor that is already present at the BBB. The immunohistochemistry staining of Fig 7 supports this idea and further shows that it is already present on the brain endothelial surface. Furthermore, as epinephrine did not alter staining intensity, this suggests that epinephrine may work more as a co-factor to active the M6P receptor rather than to affect its expression or its translocation to the cell surface. The presence of receptor that is functionally located but not activated because of the absence of alpha adrenergic stimulation may explain that while usually no uptake of I-PGUS is seen in the adult (as illustrated in Fig 1), occassionally there seems to be a measurable transport of I-PGUS in the control animals (as illustrated in Fig 6). A possibility for future studies would be to determine whether stress sufficient to elevate blood epinephrine levels could induce I-PGUS transport across the BBB. This speculation is supported by the finding that epinephrine is able to stimulate I-PGUS transport in the brain perfusion experiments (Fig 8). With brain perfusion, epinephrine comes into contact only with the luminal surface of the BBB, ruling out the possibility that epinephrine works indirectly at other tissues. It also demonstrates that the effect of epinephrine is immediate as the perfusion experiments lasted on 5 min.

These findings that epinephrine can re-induce enzyme transport is consistent with other observations that small molecules can modulate the transport of other biologics. For example, alpha1 adrenergics stimulate the transport of leptin across the BBB and the transport rate of peptide transport system-1 is modulated by several small molecules [23]. Aluminum acts as an noncompetitive modulator and leucine as a uncompetitive modulator of peptide transport system-1 [24, 25]. The rapid effect of epinephrine on M6PR transport activity is suggestive that it too is acting as an allosteric regulator.

In conclusion, we found that both alpha1 and alpha2 adrenergic receptor stimulation is required to induce I-PGUS transport across adult BBB, whereas BBB disruption is mediated by the adrenergic beta receptor. The M6P receptor is present on the cell surface of primary cultures of brain endothelial cells and I-PGUS transport is immediately induced in brain perfusion experiments where the only tissue that epinephrine can affect is the BBB. These studies show that a drug capable of stimulating both alpha1 and alpha2 adrenergic receptors as illustrated here by clonidine can induce I-PGUS transport across the BBB. An alpha adrenergic agonist free of severe side effects such as hypotension could be a useful addition to treating lysosomal storage diseases.
